# Photo- and Radiofrequency-Induced Heating of Photoluminescent Colloidal Carbon Dots

**DOI:** 10.3390/nano12142426

**Published:** 2022-07-15

**Authors:** Gauhar Mussabek, Nazym Zhylkybayeva, Ivan Lysenko, Pavlo O. Lishchuk, Saule Baktygerey, Dana Yermukhamed, Yerzhan Taurbayev, Gani Sadykov, Alexander N. Zaderko, Valeriy A. Skryshevsky, Vladyslav V. Lisnyak, Vladimir Lysenko

**Affiliations:** 1 Institute of Information and Computational Technologies, 125 Pushkin Str., Almaty 050000, Kazakhstan; naz-30@mail.ru (N.Z.); azhgireyeva@gmail.com (S.B.); danayermukhamed92@gmail.com (D.Y.); taurbayev@mail.ru (Y.T.); gani_sadikov@mail.ru (G.S.); lisnyak@univ.kiev.ua (V.V.L.); 2 Faculty of Physics and Technology, Al-Farabi Kazakh National University, 71 al-Farabi Ave., Almaty 050040, Kazakhstan; 3 Faculty of Physics, Taras Shevchenko National University of Kyiv, 64 Volodymyrska Street, 01601 Kyiv, Ukraine; ivan.lysenko305@gmail.com (I.L.); pavel.lishchuk@knu.ua (P.O.L.); 4 Corporation Science Park, Taras Shevchenko National University of Kyiv, 60 Volodymyrska Street, 01033 Kyiv, Ukraine; indeo1016@gmail.com (A.N.Z.); skryshevsky@knu.ua (V.A.S.); 5 Institute of High Technologies, Taras Shevchenko National University of Kyiv, 64/13 Volodymyrska Str., 01601 Kyiv, Ukraine; 6 Chemical Faculty, Taras Shevchenko National University of Kyiv, 64/13 Volodymyrska Str., 01601 Kyiv, Ukraine; 7 Light Matter Institute UMR-5306, Claude Bernard University of Lyon/CNRS, Université de Lyon, 69622 Villeurbanne, France; vladimir.lysenko@univ-lyon1.fr

**Keywords:** photo- and radiofrequency-induced heating, O, N-containing CDs, photoluminescent thermometry, nanocolloids for photothermal and radiofrequency therapy, photothermal effects

## Abstract

Nitrogen- and oxygen-containing carbon nanoparticles (O, N-CDs) were prepared by a facile one-step solvothermal method using urea and citric acid precursors. This method is cost-effective and easily scalable, and the resulting O, N-CDs can be used without additional functionalization and sample pretreatment. The structure of O, N-CDs was characterized by TEM, AFM, Raman, UV-vis, and FTIR spectroscopies. The obtained O, N-CDs with a mean diameter of 4.4 nm can be easily dispersed in aqueous solutions. The colloidal aqueous solutions of O, N-CDs show significant photothermal responses under red-IR and radiofrequency (RF) irradiations. The as-prepared O, N-CDs have a bright temperature-dependent photoluminescence (PL). PL/PLE spectral maps were shown to be used for temperature evaluation purposes in the range of 30–50 °C. In such a way, the O, N-CDs could be used for biomedicine-related applications such as hyperthermia with simultaneous temperature estimation with PL imaging.

## 1. Introduction

The use of fluorescent, cost-effective, and biocompatible nanoscale objects, such as carbon dots (CDs) and related carbon-based nanoparticles (CNPs), for optoelectronic and biomedical applications has been significantly rising in recent years [1,2,3]. Most synthesized CDs and CNPs have spherical or plane shapes with characteristic sizes below 10 nm. Highly fluorescent CDs codoped with diverse heteroatoms such as nitrogen, halogens, oxygen, and sulfur are widely applied as a turnoff fluorescent probe for detecting different analytes [4,5].

One of the most promising biomedical applications of CDs with sizes below 20 nm is phototherapy [6,7,8,9,10]. Indeed, CDs are known from the literature to exhibit increased photothermal conversion efficiency and to have sufficiently high visible and near-infrared (NIR) light absorbance [11]. In particular, a few reports [7,12,13] have focused on using CDs for photothermal therapy (PTT) and photodynamic therapy (PDT), which are intensively studied as efficient anticancer treatment approaches.

Along with the PTT and PDT cited above, there is another hyperthermia therapy approach based on the use of radiofrequency (RF) radiation penetrating much deeper than VIS-NIR light in biological tissues and inducing their local temperature rise [14]. For example, radiofrequency electromagnetic fields have been shown to induce thermal toxicity in malignant cells [15,16,17]. A RF device operating at 13.56 MHz was reported to heat single-walled carbon nanotubes (SWCNTs) in view of their application for cancer treatment [18]. In this study, SWCNTs internalized in human cancer cells were exposed to RF radiation to induce their noninvasive, selective, concentration-dependent thermal destruction. Direct intratumoral injection of SWCNTs followed by the RF treatment for 48 h led to complete necrosis of the tumors when compared to the controls. However, to the best of our knowledge, there are still no reports on the RF-induced heating of CDs.

One of the important issues for the application of carbon-based nanomaterials for photo- and RF-induced hyperthermia is contactless temperature control. CDs are known to have temperature-dependent fluorescence spectra [18,19]. Thus, one could use spectral fluorescence features to estimate temperature in places where CDs are localized. Due to their outstanding properties, including intense fluorescence, photobleaching resistance, chemical stability, low-cost precursors, low toxicity, and enhanced biocompatibility [18,19,20,21,22], CDs were used as temperature-sensing materials with a high spatial resolution at the nanoscale level. Recent reports [23,24,25,26] showed the thermal-sensing behavior of CDs for a better understanding of biological processes.

The primary objective of our present study is to find whether the O, N-containing fluorescent CDs (O, N-CDs) can act as multimodal nanomaterials for photo- and RF-induced heating with temperature estimation from their fluorescent spectra. In particular, the main idea of our study is to show that the heating of a CD-based colloidal solution by means of RF or NIR electromagnetic waves can be efficiently controlled by the estimation of temperature in physiological (30–38 °C) and therapeutic (38–46 °C) ranges. Such temperature control will allow avoiding tissue overheating and thus preventing any undesirable necrotic effects.

## 2. Materials and Methods

### 2.1. Synthesis of CDs

Strongly fluorescent O, N-CDs were synthesized by the solvothermal carbonization method using urea and citric acid as described in detail in one of our previous papers [27]. The obtained O, N-CDs were purified by resedimentation with hydrochloric acid to remove organic species originating from synthesis products. In particular, chromato–mass spectra analysis allowed us to conclude an absence of any low-molecular fluorophores in the final colloidal solutions of O, N-CDs (Figure S1 in Supplementary Materials). Concentration of O, N-CDs in the aqueous colloidal solutions was adjusted to be 0.625 g/L as confirmed by gravimetry.

### 2.2. Instrumentation for Characterization

Morphology and microstructure of O, N-CDs were characterized by means of transmission electron microscopy (TEM, Jeol JEM-2100F, Tokyo, Japan, 200 kV) and atomic force microscopy used in tapping mode (AFM, Integra Prima Basic, NT-MDT LCC, Moscow, Russia). UV-visible absorption spectra were recorded with an UV-visible spectrophotometer (UV-2700, Shimadzu, Kyoto, Japan). Raman spectrum of O, N-CDs deposited on a previously cleaned Si wafer was collected with an integrated confocal micro-Raman system (LabRAM ARAMIS μ-Raman spectrometer, Horiba-Jobin Yvon, Inc., Edison, NJ, USA) equipped with a diode-pumped 633 nm solid-state laser [27]. Fourier-transform infrared attenuation reflection (FTIR ATR) spectrum was recorded with an IR spectrometer (Prestige-21, Shimadzu, Kyoto, Japan) equipped with an ATR accessory (MIRacle, PIKE Technologies, Fitchburg, WI, USA). Zeta potential (ZP) of a colloid solution of O, N-CDs was measured at 21 °C with a particle size and zeta potential analyzer (Zetasizer Nano analyzer, Malvern Pananalytical, Malvern, UK).

### 2.3. Photoinduced Heating

To perform photoinduced heating of 1.7 mL colloidal solution of O, N-CDs in a 4 mL quartz cuvette; 650 nm (red) and 850 nm (NIR) laser diode modules were used. Laser beams were focused in the center of the cuvette containing the colloidal solution. The lasers operated in continuous wave mode. Optical powers of the laser beams were measured to be 125 mW and 680 mW for the red and NIR laser, respectively. To maintain the radiation wavelength and output power with high accuracy, the laser-controlling modules were equipped with a laser diode thermal stabilization system (see photo of experimental set-up in Figure S2 in Supplementary Materials). Concentration of the O, N-CDs in the colloid aqueous solution was 0.625 g/L. Temperatures of aqueous suspensions of O, N-CDs and pure water (for comparison) were measured with an IR thermometer (CTlaser LT, Optris GmbH, Berlin, Germany).

### 2.4. RF-Induced Heating

The RF heating was produced using a medical ultra high frequency apparatus (UHF-60, MedTeCo, Moscow, Russia), which is commonly employed for the RF physiotherapy. The apparatus provided RF radiation with a frequency of 27.12 ± 0.163 MHz and power values between 30 and 50 W. The apparatus contained a power source coupled to a pair of flat electrodes with a diameter of 50 mm and fixed distance of 10 mm between them. Temperatures of aqueous suspensions of O, N-CDs and pure water (for comparison) were measured with an IR thermometer (CTlaser LT, Optris GmbH, Berlin, Germany). To investigate the RF heating process, a 4 mL quartz cuvette was filled with 2 mL of O, N-CDs-based colloids (as shown in Figure S4 in Supplementary Materials). Concentration in the colloidal suspensions of O, N-CDs was 0.625 g/L.

### 2.5. Fluorescence Measurements

Stationary fluorescence spectra (excitation/emission maps) of 2 mL colloidal solution of O, N-CDs in a 4 mL quartz cuvette were recorded on a spectrofluorometer (RF-6000, Shimadzu, Kyoto, Japan) with a 150 W xenon arc lamp as the excitation source, as well as with the possibility of temperature control.

## 3. Results and Discussion

### 3.1. Structure of O, N-CDs

**Figure 1:**
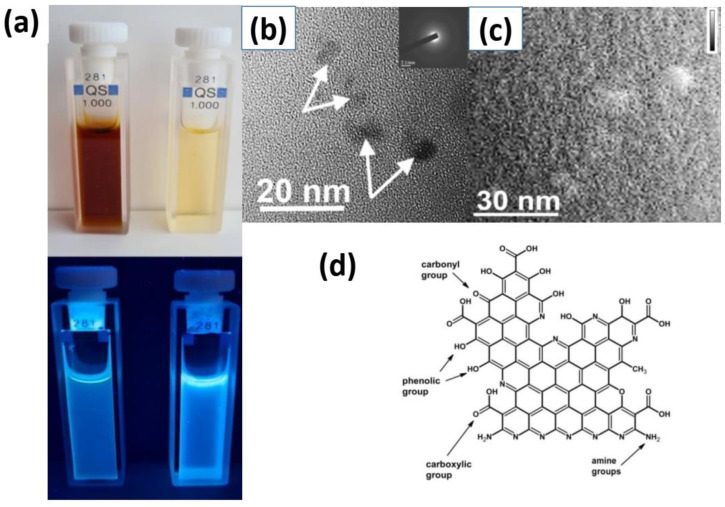
(**a**) Photos of water solutions with dispersed O, N-CDs in daylight and under the UV-light illumination. (**b**) TEM image of O, N-CDs (insert SAED pattern). The O, N-CDs are shown with arrows. (**c**) AFM images showing the topographic view of the O, N-CDs. (**d**) Schematic structure of a single O, N-CD adapted from [27].

The synthesis of O, N-CDs derived from the thermal processing of urea with citric acid precursors has been already previously reported in detail by our group [27,28]. Figure 1a shows pictures of the O, N-CDs-based colloidal solutions under daylight and UV excitation. By varying heating regimes, one can obtain O, N-CDs with sizes from 4 to 7 nm. The shape and structure of the O, N-CDs were studied by means of TEM (see Figure 1). In particular, statistical analysis of 100 particles captured from the TEM images of the obtained O, N-CDs gave a mean diameter of a single CD to be about 4.4 ± 0.6 nm (Figure 1b) and about 16.6 nm of their aggregates according to the AFM image shown in Figure 1c. It should be noted that the diffraction contrasts of the O, N-CDs are very low, and the lattice fringes are absent on the high-resolution TEM images (see insert in Figure 1b). This fact indicates a rather amorphous structure of the O, N-CDs cores. According to the FTIR spectroscopy and XPS data (see Figure S5 in Supplementary Materials) [27], the surface of O, N-CDs is predominantly oxidized. The zeta potential (ZP) value of the O, N-CDs representing their superficial electrical charge was found to be about −34.4 mV.

Earlier reported XPS and FTIR ATR spectral data [27] confirm a possible chemical composition of the O, N-CDs as sketched in Figure 1c. Amino groups originated from the urea used in the solvothermal synthesis of the O, N-CDs partially conserve their nature, namely, their weak ability to be protonated. Stretching vibration peaks of NH- and CH-groups and different OH-groups are clearly visible on the FTIR-ATR spectrum of the O, N-CDs (Figure S5 Supplementary Materials). Selected vibrations of =N---H and C=N groups originate from nitrogen atoms incorporated in the structure of O, N-CDs.

The Raman spectra of the studied O, N-CDs indicate an absence of clear ordering due to the high amorphicity of the synthesized CDs (see Figure S5 in Supplementary Materials). The absorbance spectra of the O, N-CDs measured in the UV-visible spectral range exhibit characteristic peaks for this kind of CD that mainly correspond to *n*→π* and π→π* electron transitions in the conjugated carbonyl groups (C=C−C=O) and C=C bonds [27] (see Figures S6 and S7 in Supplementary Materials).

### 3.2. Photoinduced Heating of CDs-Based Colloids

**Figure 2:**
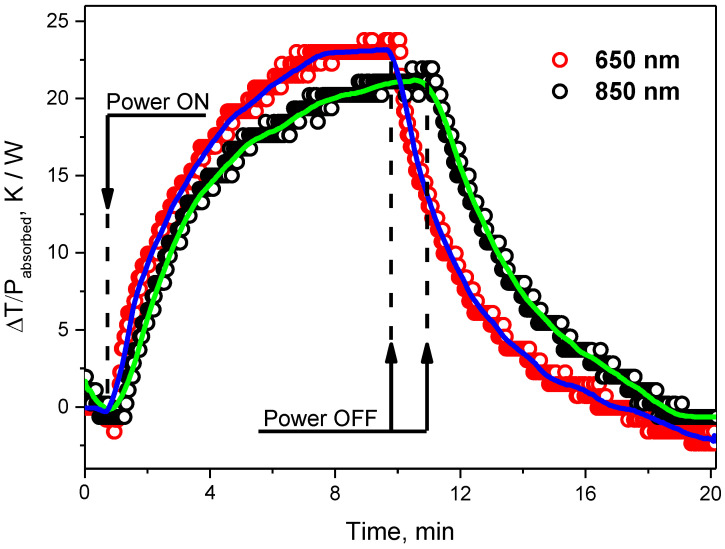
Photoinduced heating/cooling of colloidal solutions of O, N-CDs in water at two laser wavelengths: 650 and 850 nm. Open symbols correspond to experimental points and solid lines to exponential-like fitting curves.

The photothermal (PT) effect induced by a laser beam and leading to heating of the colloidal solutions of the O, N-CDs can be seen in Figure 2. The temperature rise per unit of absorbed laser power (ΔT/P) reaches 23 and 22 K W^–1^ for 9 min and 11 min of heating at the laser radiation wavelength of 650 and 850 nm, respectively. Both laser wavelengths (red and near infrared) were chosen because of their frequent use for therapeutics purposes. The slightly higher PT effect is achieved for the lowest irradiation time for the red-exciting laser (650 nm) because of the higher absorption of O, N-CDs at this wavelength. Taking into account the data shown in Figure 2, a laser can easily heat the colloidal solution to 20 °C above the surrounding temperature. To reach any required temperature, one can adjust the exposure time and/or power of the laser radiation.

Contrary to bulk semiconductors, for which the PT effect is mainly due to band–band absorption followed by the thermalization/recombination of free charge carriers, the photoinduced heating of colloidal molecular-like CDs occurs due to the absorption of the laser radiation (650 nm and 850 nm) leading to electron transition via electronic states corresponding to core and surface chemical bonds. As one can see on the absorption spectra of O, N-CDs in the red–near infrared spectral range (Figure S8 in Supplementary Materials), their absorption coefficients at 650 nm are higher than those at 850 nm because of the higher density of electronic states at shorter wavelengths.

The data represented in Figure 2 allowed the evaluation of a photothermal conversion efficiency *η* for the studied O, N-CDs-based colloidal solutions from the following equation:

(1) $$\eta =\frac{hA\left(T_{max}-T_{surr}\right)-Q_{Dis}}{I\left(1-10^{-A\lambda }\right)}$$

where *h* is the heat transfer coefficient, *A* is the surface area of the container, and *T_max_* and *T_surr_* are the equilibrium temperature and the ambient temperature, respectively. *Q_Dis_* expresses the heat dissipation from the light absorbed by the quartz cuvette, which is 37.8 mW. *I* is the incident laser power which was 3.98 W/cm^2^ and 21.65 W/cm^2^ for red and IR lasers, respectively. *Aλ* is the absorbance of the O, N-CDs at 650 nm (0.132) and 850 nm (0.028).

The value of *hA* can be calculated according to the approach described in [10]:

(2) $$hA=-\frac{m_{w}c_{w}\ln\left(\frac{\Delta T}{\Delta T_{max}}\right)}{t}\;\;\;$$

where *m_w_* and *c_w_* are the mass (0.17 g) and heat capacity (4.2 J/g) of deionized water used as a solvent, respectively. ∆*T* is the temperature change, a temperature difference between the solution temperature and ambient temperature. ∆*T_max_* is the temperature change at the maximum steady-state temperature; *t* is the linear time range from the cooling part of the curves. In our experiments, the photothermal conversion efficiencies *η* of the O, N-CDs were found to be 35.8% and 27.8% for heating induced by 650 nm and 850 nm lasers, respectively.

### 3.3. RF-Induced Heating of CDs-Based Colloids

RF-induced heat generation near a solid-state or molecular-like nanoparticle (NP) surrounded by an electrolyte solution is already a well-known physical phenomenon [29,30]. For example, a model, explaining strong heating of weakly conductive Si-based NPs induced by RF radiation, has been proposed in [30]. The electrical double layer at the NP/solution interface is rapidly and continuously polarized due to its exposure to the oscillating electric field of RF radiation. According to the model, the Joule heating is due to local alternative electrical currents occurring around the polarized NPs/solution interface. The heating rate can be estimated by considering the Joule heating in the electrical double layers according to the following relation:

(3) $$\frac{dT}{dt}=\frac{NW}{c\rho }=\frac{NE^{2}d}{c\rho }$$

where *N* is the NPs concentration, *W* is the heat power released per an NP, *E* is the RF electric field strength, *d* is the diameter of NPs, and c and *ρ* are the heat capacity and density of water, respectively.

**Figure 3:**
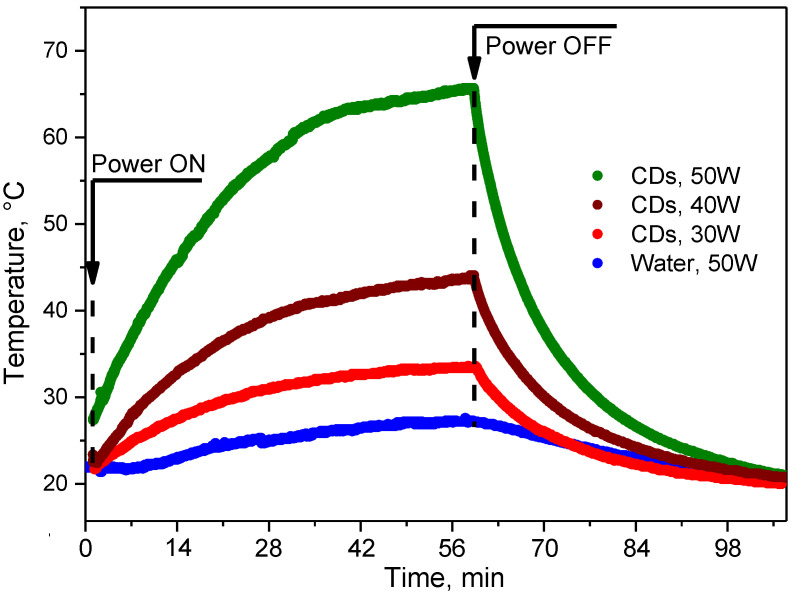
RF-induced heating/cooling of colloidal solutions of O, N-CDs in water at the RF generator powers of 50, 40, and 30 W and water at the RF generator power of 50 W.

Considering the spherical-like O, N-CDs with a mean diameter of 4.4 ± 0.6 nm studied in the present work, the mechanism of RF-induced heating is similar to the case of the Si-based colloids as mentioned above. Despite the low electrical conductivity of the O, N-CDs, these NPs showed prominent RF-induced heating rates. Indeed, based on the experimental data shown in Figure 3, it is possible to estimate a maximum heating rate corresponding to the initial linear part of the time-dependent temperature evolution curves. In particular, such heating rates in our experiments were: 0.50, 0.90, and 1.55 °C min^–1^ for the RF radiation powers of 30, 40, and 50 W, respectively (see Figure 3). The maximum heating rate achieving the value of 1.55 °C min^–1^ is more than 10 times higher than that for deionized water (0.13 °C min^–1^). Thus, at these experimental conditions, one can easily reach any temperature values from the therapeutic range of 40–50 °C.

### 3.4. Thermometry with Fluorescent CDs-Based Colloids

The temperature level resulting from the photo- and RF-induced heating of O, N-CDs-based colloids has to be controlled as precisely as possible. A special IR thermometer was used in the experiments reported above. However, to avoid the use of any external device, one can perform the temperature measurement based on the temperature-dependent fluorescence of the CD colloidal solutions. For example, N-doped CDs with a fluorescence maximum corresponding to 502 nm were reported to be a function of temperature in the range between 25 °C and 95 °C [31]. Usually, the fluorescence intensity of CDs decreases with a rise in temperature [21].

**Figure 4:**
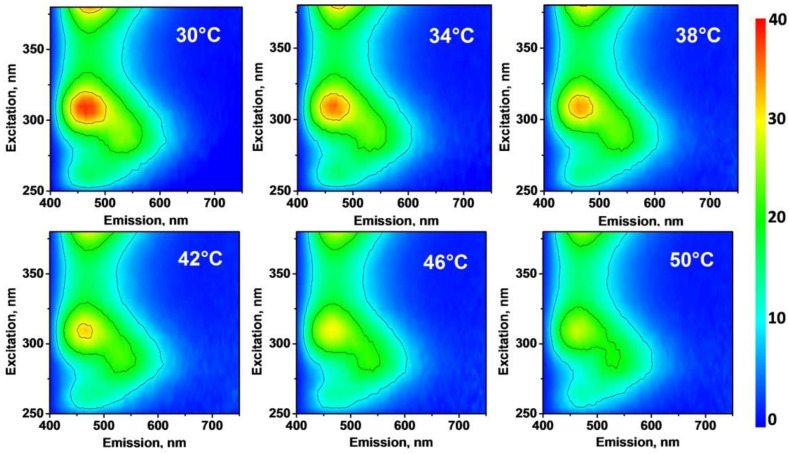
Temperature-dependent PL excitation/emission maps of colloidal solution of O, N-CDs in water at 0.625 g/L.

**Figure 5:**
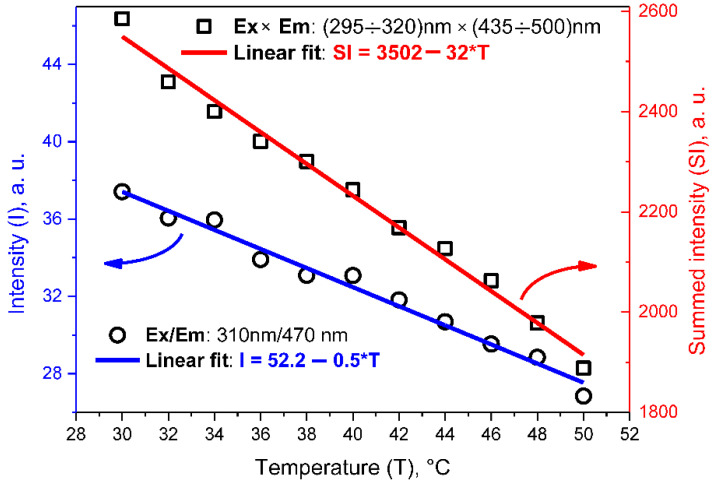
Temperature-dependent PL intensity of colloidal solutions of O, N-CDs in water (0.625 g/L). Black symbols—experimental points. Blue and red lines are linear fits of the experimental points corresponding, respectively, to a single spectral point Exc/Em: 310/470 nm and a spectral area: Exc × Em: (295–320) nm × (435–500) nm.

In the present study, the temperature-dependent evolution of the PL/PLE maps shown in Figure 4 has been studied. As one can see, the temperature increase is accompanied by a monotonous decrease in the overall PL intensity for any exciting wavelength from the range: 250–380 nm. In particular, a clear reduction in the PL intensity as a function of rising temperature can be observed in the relatively large spectral range: 400–550 nm. Such a temperature dependence of the PL intensity can be explained by the more important role of nonradiative recombination channels (involving various defect and surface electronic states) at higher temperatures [32,33,34,35,36]. Moreover, perfect linear dependences of the PL intensities on temperature are illustrated in Figure 5 for a single spectral point as well as for a chosen spectral area. Thus, the PL/PLE spectra of O, N-CDs can be used for the evaluation of temperature in the physiological and therapeutic temperature ranges corresponding to 30–40 °C and 40–50 °C, respectively.

## 4. Conclusions

The N, O-containing CDs exhibited interesting unique photothermal properties to be exploited in photo- and RF-induced hyperthermia. We demonstrated that the N, O-containing CDs exhibit notable PT responses. In particular, the PT effect was quantified as a function of irradiating power under photo- and RF-induced heating. A facile preparation protocol and enhanced biocompatibility of the N, O-containing CDs can help to pave the way for their variety of biomedical applications. In particular, the PT effect observed under NIR and RF heating suggests that the N, O-CDs might have a high potential for combined in vivo PL imaging and PT therapy.

## Supplementary Materials

The following supporting information can be downloaded at: https://www.mdpi.com/article/10.3390/nano12142426/s1, Figure S1: (a,b) Chromato-mass traces for the (O, N)-CDs; Figure S2: (a,b) UHF-60 device: 1—the electronic unit, 2—control buttons, 3—display, 4—setting indicator, 5—electrode holder fixing screws, 6—electrodes, 7—electrode holders, 8—colloidal solution, 9—IR thermometer Optris CTlaser LT; Figure S3: Experimental set-up for the photo-induced heating of a colloidal solution of (O, N)-CDs: 1—a cuvette with the colloidal solution, 2—the IR thermometer Optris CTlaser LT, 3—laser 1, 4—laser 2; Figure S4: “ИMO-2H” device for the measurements of laser power; Figure S5: FTIR-ATR spectrum of (O, N)-CDs; Figure S6: Raman spectrum of studies (O, N)-CDs; Figure S7: UV-Vis absorbance spectrum of (O, N)-CDs; Figure S8: Near IR absorbance spectrum of (O, N)-CDs. References [27,37,38,39,40,41,42,43,44,45,46,47,48,49,50,51,52,53,54,55,56,57,58] are cited in the supplementary materials.
