# Supplementary material for: Photo- and Radiofrequency-Induced Heating of Photoluminescent Colloidal Carbon Dots
Source: Nanomaterials (Basel). 2022 Jul 15;12(14):2426. doi: 10.3390/nano12142426 (PMC9324485; doi:10.3390/nano12142426)
Supplement: Supplementary file 1 [file nanomaterials-12-02426-s001.zip › nanomaterials-1772202-supplementary.pdf]

Supplementary materials

Photo- and radiofrequency-induced heating of photoluminescent colloidal carbon dots

Gauhar Mussabek[a, b, *], Nazym Zhylkybayeva[a,b], Ivan Lysenko[c], Pavlo O. Lishchuk [c, d], Saule Baktygerey[a, b], Dana Yermukhamed[a, b], Yerzhan Taurbayev[a, b], Gani Sadykov[a], Alexander N. Zaderko[d, f], Valeriy A. Skryshevsky[d, f], Vladyslav V. Lisnyak[a, e] and Vladimir Lysenko[g]

[a] Institute of Information and Computational Technologies, 125 Pushkin Str., Almaty, 050000, Kazakhstan
[b] Al-Farabi Kazakh National University, 71 Al-Farabi Ave., Almaty, 050040, Kazakhstan
[c] Faculty of Physics, Taras Shevchenko National University of Kyiv, 64 Volodymyrska Street, Kyiv, 01601, Ukraine
[d] Corporation Science Park, Taras Shevchenko University of Kyiv, 60 Volodymyrska Str., Kyiv, 01033, Ukraine
[e] Institute of High Technologies, Taras Shevchenko National University of Kyiv, 64/13 Volodymyrska Str., Kyiv, 01601, Ukraine
[f] Chemical Faculty, Taras Shevchenko National University of Kyiv, 64/13 Volodymyrska Str., Kyiv, 01601, Ukraine

[g] Light Matter Institute UMR-5306, Claude Bernard University of Lyon/CNRS, Université de Lyon, Villeurbanne, 69622, France

*Correspondence: Mussabek, G.; Institute of Information and Computational Technologies, 125 Pushkin Str., Almaty, 050000, Kazakhstan; Al-Farabi Kazakh National University, 71 Al-Farabi Ave, Almaty, Kazakhstan; email: gauhar.musabek@kaznu.kz

Table of content

Chromato-mass traces for the (O, N)-CDs..……..…………..S1
UHF-60 device ………………………………………… S2
Experimental set-up for the photo-induced heating ………… S3
A “ИМО-2Н” device………………………………………S4
FTIR-ATR spectrum of (O, N)-CDs..……………………S5
Raman spectrum of studies (O, N)-CDs..…………..………S6
UV-Vis absorbance spectrum of (O, N)-CDs…………..…S7

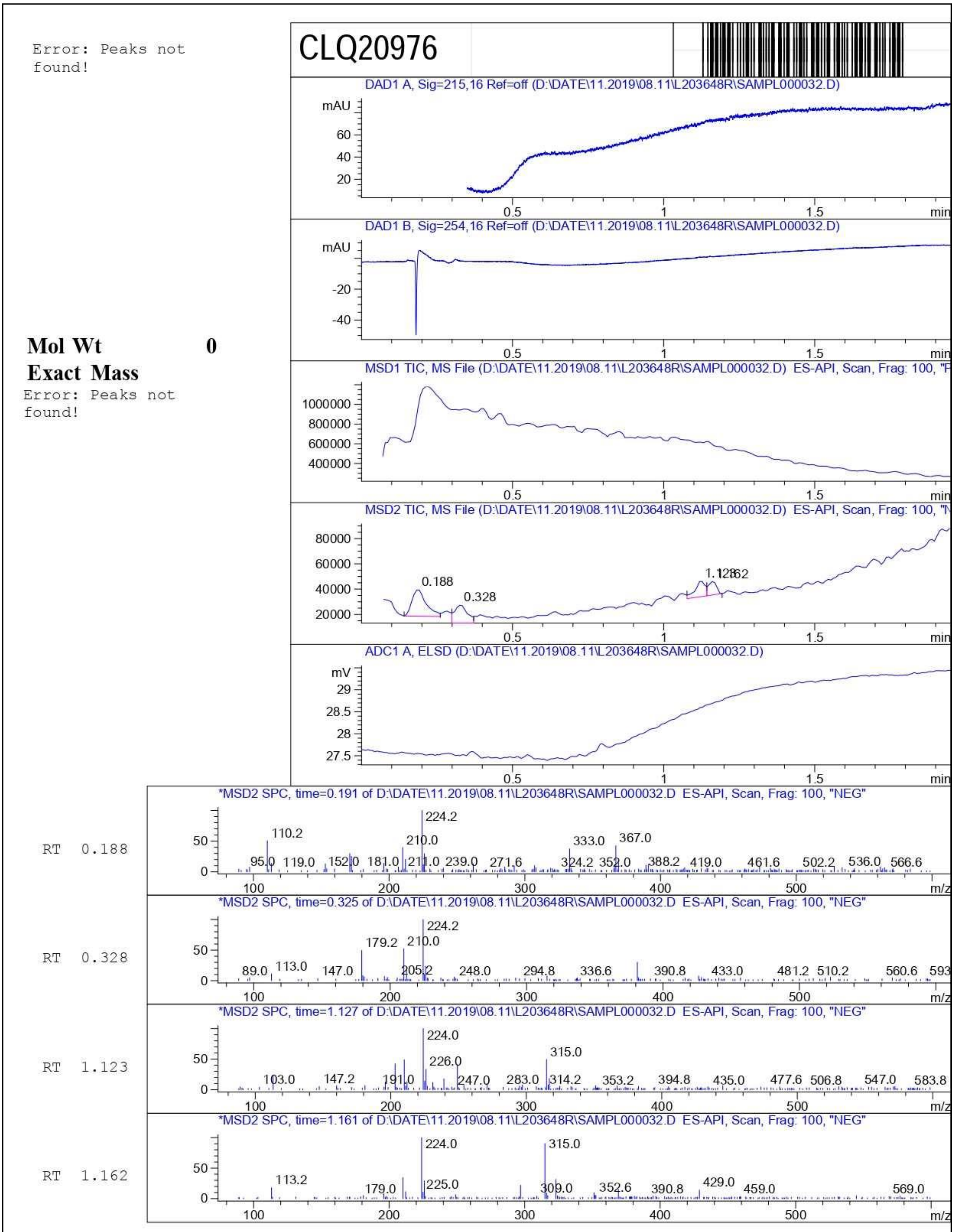
Error: Peaks not found!
CLQ20976
DAD1 A, Sig=215,16 Ref=off (D:\DATE\11.2019\08.11\L203648R\SAMPL000032.D)
DAD1 B, Sig=254,16 Ref=off (D:\DATE\11.2019\08.11\L203648R\SAMPL000032.D)
Mol Wt 0
Exact Mass
Error: Peaks not found!
MSD1 TIC, MS File (D:\DATE\11.2019\08.11\L203648R\SAMPL000032.D) ES-API, Scan, Frag: 100,
MSD2 TIC, MS File (D:\DATE\11.2019\08.11\L203648R\SAMPL000032.D) ES-API, Scan, Frag: 100,
ADC1 A, ELSD (D:\DATE\11.2019\08.11\L203648R\SAMPL000032.D)
RT 0.188
*MSD2 SPC, time=0.191 of D:\DATE\11.2019\08.11\L203648R\SAMPL000032.D ES-API, Scan, Frag: 100, "NEG"
RT 0.328
*MSD2 SPC, time=0.325 of D:\DATE\11.2019\08.11\L203648R\SAMPL000032.D ES-API, Scan, Frag: 100, "NEG"
RT 1.123
*MSD2 SPC, time=1.127 of D:\DATE\11.2019\08.11\L203648R\SAMPL000032.D ES-API, Scan, Frag: 100, "NEG"
RT 1.162
*MSD2 SPC, time=1.161 of D:\DATE\11.2019\08.11\L203648R\SAMPL000032.D ES-API, Scan, Frag: 100, "NEG"

(a)

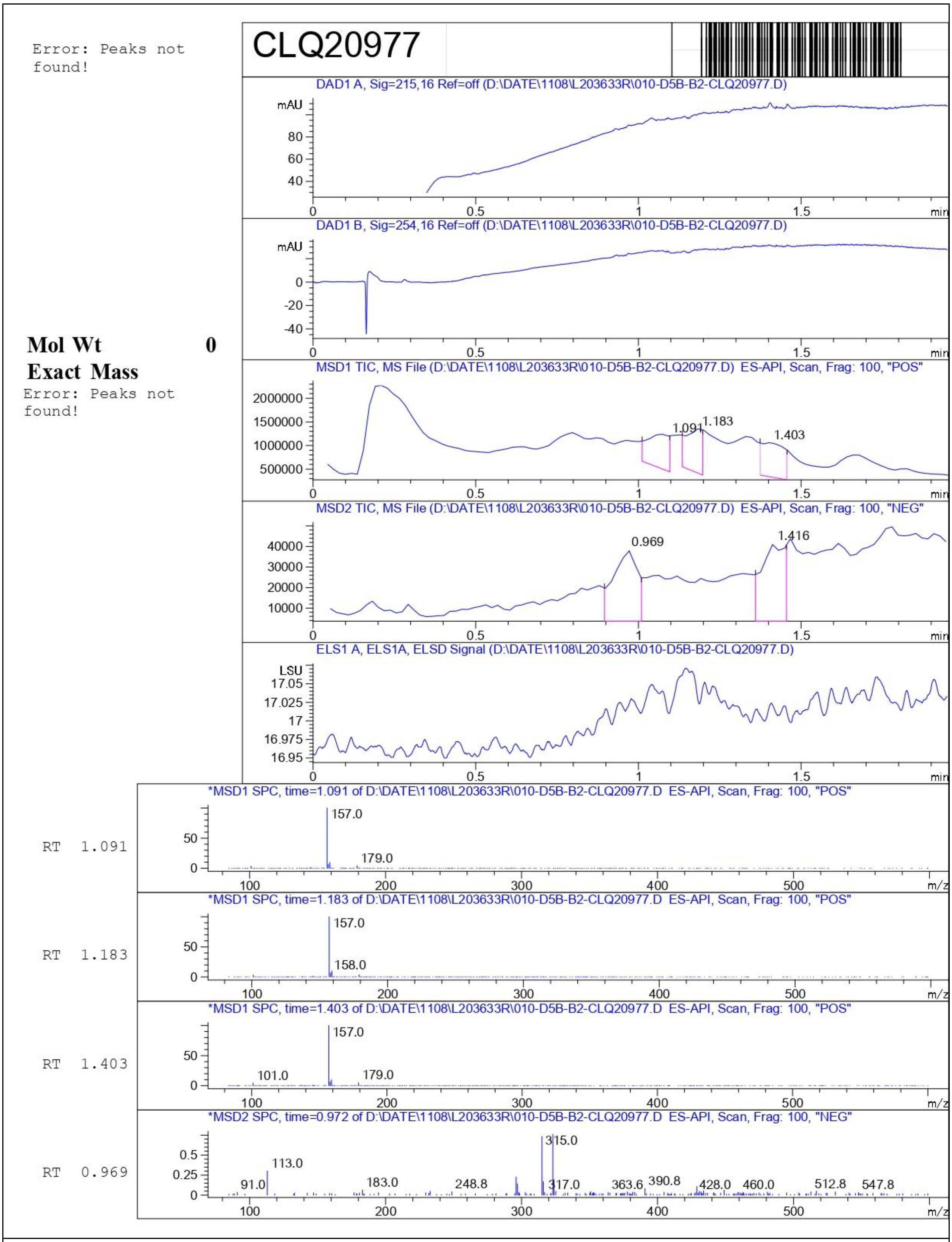

(b)

Figure S1. (**a**, **b**) Chromato-mass traces for the (O, N)-CDs.

The separation uses the same method as the protein fractionation, but there are limitations due to the large range of molecular weight values, deviations from the spherical shape, differences in the

quaternary structure of the aggregates of CDs, significant negative charge on their surface in the neutral pH region. In our opinion, as a first approximation, we can say that the first results of chromato-mass spectroscopy indicate that the synthesis results in the formation of a category of chemicals with a wide range of molecular weights, which is difficult to separate by chromatographic methods.

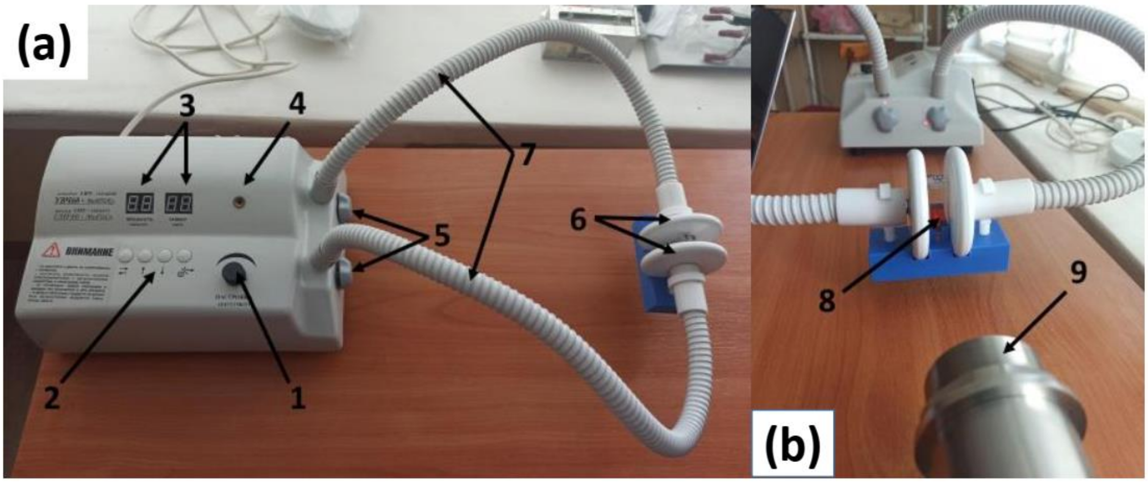

Figure S2. (**a**, **b**) UHF-60 device: 1 – the electronic unit, 2 – control buttons, 3 – display, 4 – setting indicator, 5 – electrode holder fixing screws, 6 – electrodes, 7 – electrode holders, 8 – colloidal solution, 9 – IR thermometer Optris CTlaser LT.

The device UHF-60 (fabricated by MedTeCo) is designed for local therapeutic exposure to a high-frequency electromagnetic field with the operating frequency of 27.12±0.163 MHz and the adjustable output power values of 10–60 W.

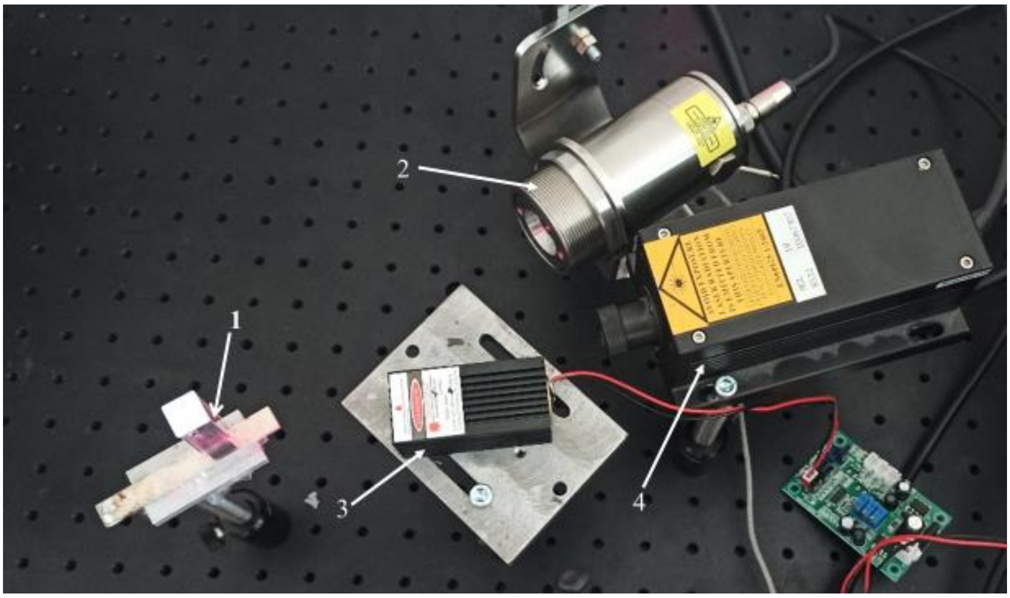

Figure S3. Experimental set-up for the photo-induced heating of a colloidal solution of (O, N)-CDs: 1 – a cuvette with the colloidal solution, 2 – the IR thermometer Optris CTlaser LT, 3 – laser 1, 4 – laser 2.

In the photo-induced heating experiments two lasers were used: (i) 650 nm, 125 mW, and (ii) 850 nm, 680 mW, see Fig. S3. Laser powers were checked by means of a "ИМО-2Н" device shown below in Fig. S4.

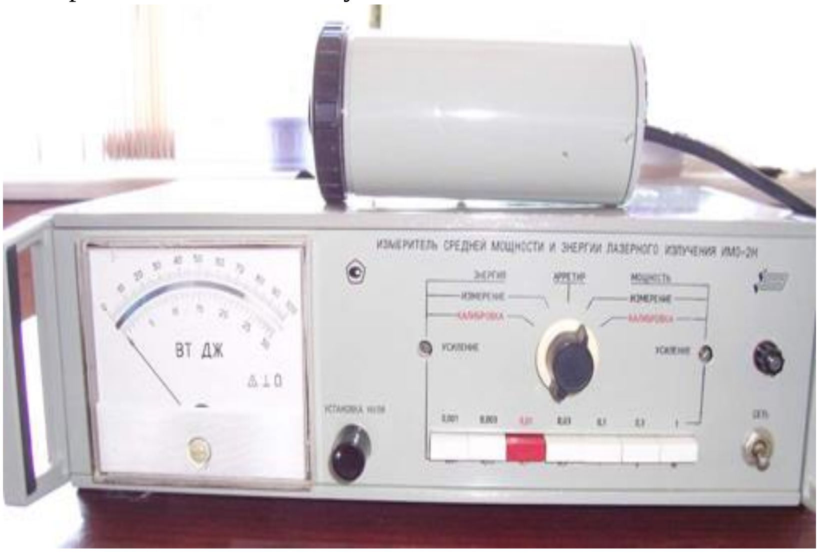

Figure S4. A "ИМО-2Н" device for the measurements of laser power.

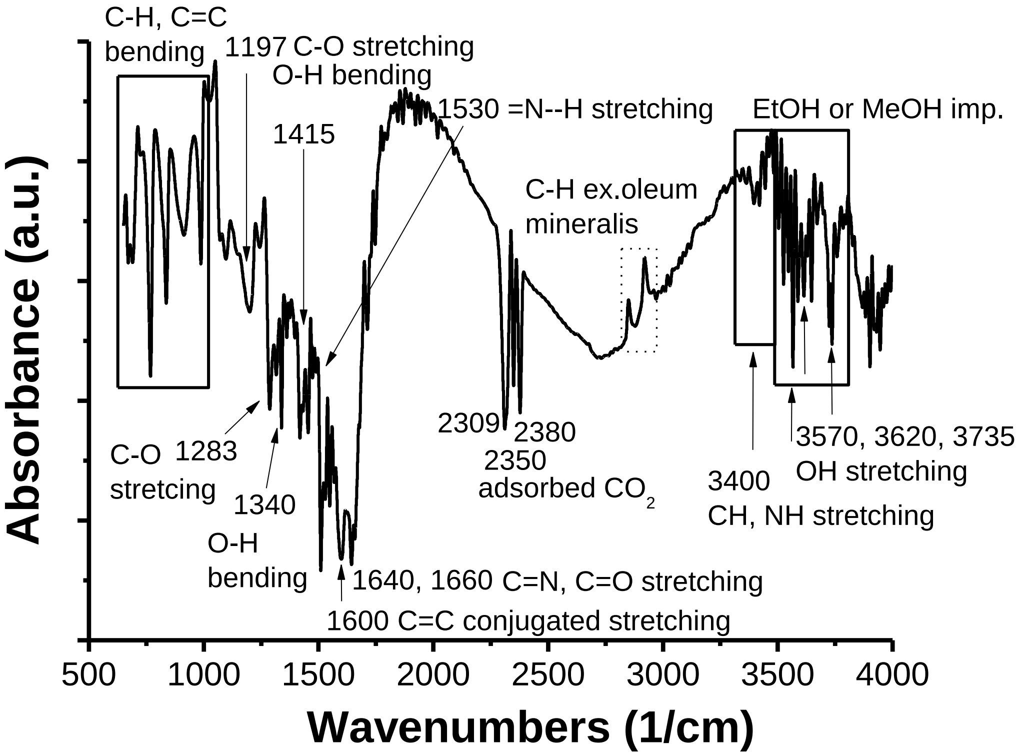

Figure S5. FTIR-ATR spectrum of (O, N)-CDs.

Figure S5 shows the FTIR-ATR spectrum of (O, N)-CDs similar to that reported earlier in [27]. One can see that stretching vibration peaks of NH- and CH-groups at 3364–3457 cm^{-1} and different OH-groups at 3503–3818 cm^{-1} are presented in the FTIR-ATR spectrum of the (O, N)-CDs. There are also strong, stretching vibration bands peaked at 1415 cm^{-1}, 1530 cm^{-1}, 1600 cm^{-1}, 1640 cm^{-1} and 1662 cm^{-1}, ascribed to the vibrations of O–H, =N---H, C=C, C=N, and C=O bonds, respectively. The selected =N---H and C=N vibration indicate that the nitrogen is incorporated in the (O, N)-CDs.

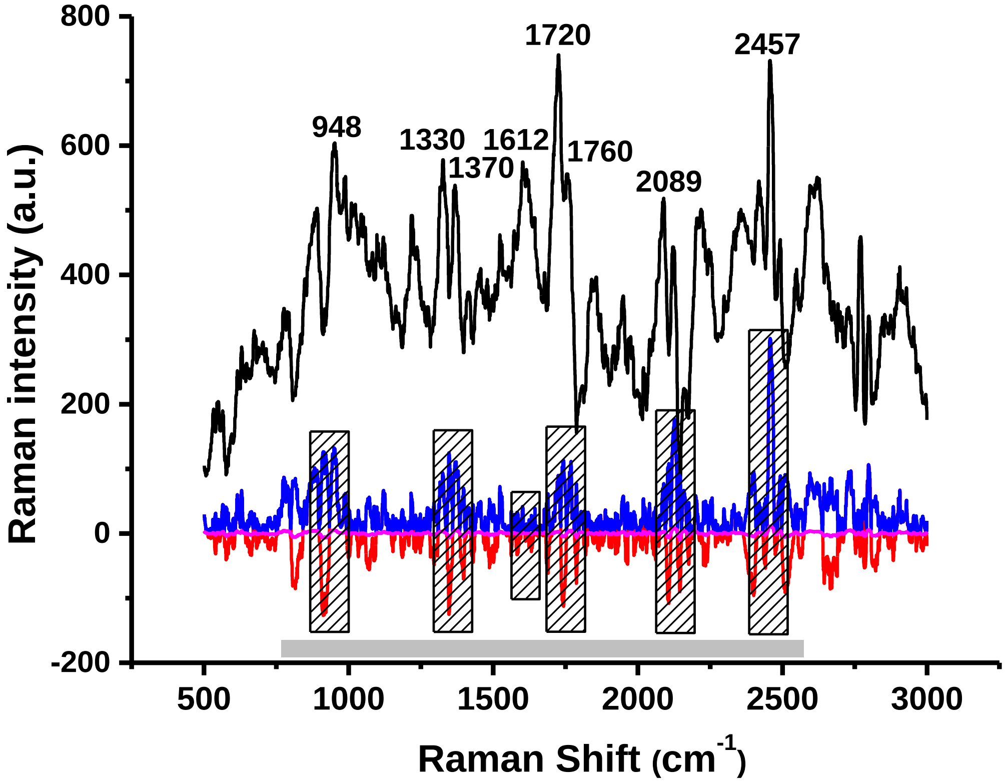

Figure S6. Raman spectrum of studies (O, N)-CDs.

As shown in Fig. S6, the Raman spectrum, as an asymmetric halo, arises due to a laser-induced fluorescence and an absence of clear structuring, as in graphene or graphite [27, 37], because of the high amorphisity of the (O, N)-CDs. After the Savitzky-Golay smoothing and differentiation filter, we obtained a set of second-derivative bands. According to Fig. S6, the main Raman lines of the O, N)-CDs spectrum have peaks at 948 cm^{-1}, 1330 and 1370 cm^{-1}, 1612 cm^{-1}, 1720 cm^{-1}, 1760 cm^{-1}, 2089 cm^{-1}, and 2457 cm^{-1} are assigned to the vibrational modes of the C–H bond in the aromatic ring in the carbon(N) skeleton, the combination of C–C and C–O, or C–C and C–N bonds [38, 39], the deformation of the aromatic ring, and the C–N–C bonds, the C=O stretching in COOH groups attached to the heterocyclic structure, and the adsorbed CO_2 stretching [40], respectively. Strong defect-induced bands at 1330 and 1370 cm^{-1} can confirm the successful incorporation of N atoms into the carbon matrix [41, 42].

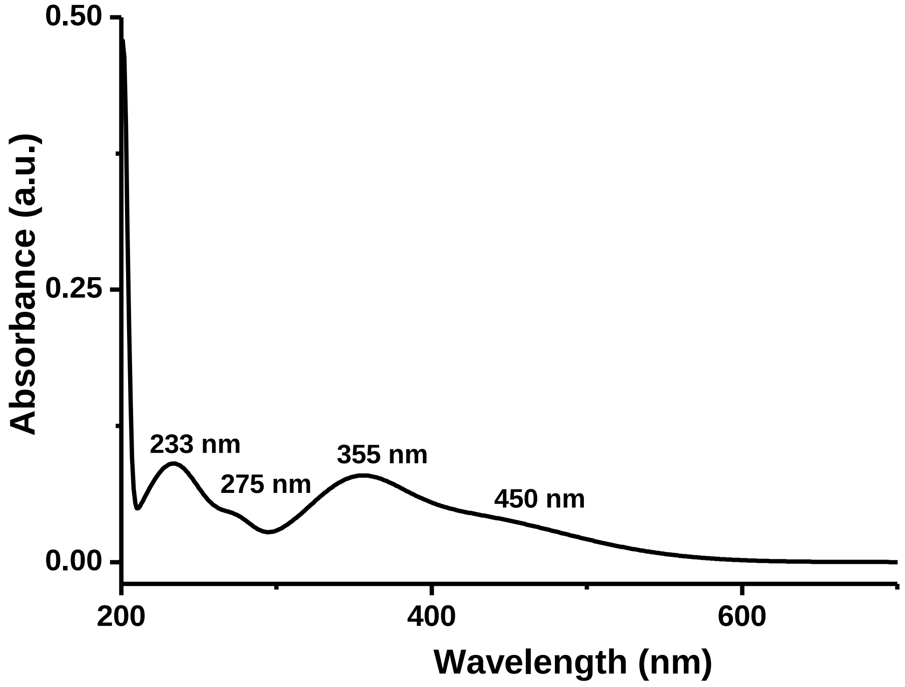

Figure S7. UV-Vis absorbance spectrum of (O, N)-CDs.

The UV-Vis absorption spectrum of a 0.625 g/L water solution of (O, N)-CDs was recorded at room temperature in a UV-2700 Multipurpose UV-Visible Spectrophotometer (Shimadzu, Kioto, Japan) within the 200 to 600 nm range in a quartz cuvette and with ~1 nm of spectral resolution. The SpectraGryph software [43] was used for the initial treatment of the spectrum. After that, the Fityk open-source software [44] was applied for the baseline handling, nonlinear spectrum fitting, and deconvolution of the resulting absorption spectrum.

The UV–Vis absorption spectrum of the (O, N)-CDs shows strong optical absorption in the UV region with a tail extending out into the visible range, as reported for N-containing CDs in Refs. [45, 46]. First, the absorbance peak of the (O, N)-CDs is at approximately 233 nm, which has resulted from the π–π^* transition [47]. On the other hand, the absorbance peak shoulder is centered at 275 nm approximately. Second, there is a noticeable absorbance increase of (O, N)-CDs from 300 to 450 nm [48]. The absorption peaks at 275 and 355 nm are like those in the UV-Vis absorption spectra of CDs reported in [49, 50]. In addition, fluorescence analyses in Fig. 4 display about the same range of an excitation-dependent PL emission with the same maximum excitation and emission wavelengths.

The specific assignment of the spectral peaks was given in Refs. [49-58]. Two peaks can be ascribed to C=C (~233 nm) bonds in the aromatic sp^2 domains and C=O (~355 nm) bonds, according to Refs. [49, 51]. There are also absorption peaks of C=N (275 nm), C=C=O (450 nm), and a tail in the low-energy region (>500 nm) [49, 51]. Compared with N-free CDs, the optimal N-CDs [52–57] and the studied (O, N)-CDs exhibit a much intensive absorption peak around 335 nm and a more broad absorption band extending to 550 nm (Fig. S7). Without a doubt, the absorption peak at 335 nm corresponds to the n–π^* transition of C=O bonds. The visible absorption band can be attributed to the amino groups on the surface of N-CDs as in Refs. [53, 57]. The expanding absorption to 550 nm indirectly explains the nitrogen-doping of CDs [58]. Assigned as in Refs. [54-56, 58], the absorption peaks located at below 320 nm could be from the electron π–π^* transitions of C=C, C=N, and C=O groups.

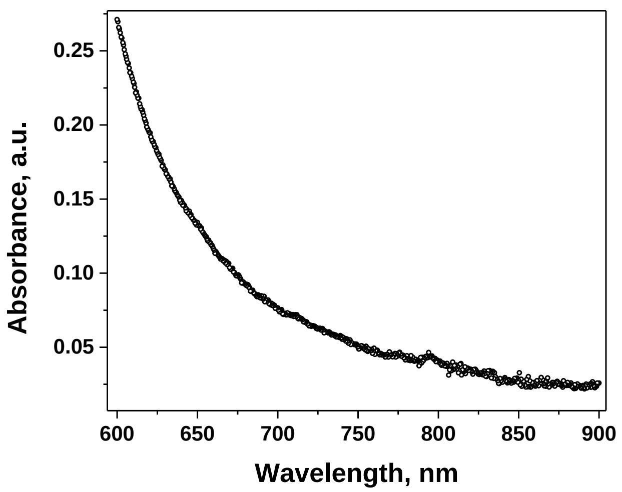

Figure S8. Near IR absorbance spectrum of (O, N)-CDs.

References

[27] Ivanov, I.I.; Zaderko, A.N.; Lysenko, V.; Clopeau, T.; Lisnyak, V.V.; Skryshevsky, V.A. Photoluminescent recognition of strong alcoholic beverages with carbon nanoparticles. *ACS Omega* **2021**, *6*, 29, 18802–18810. https://doi.org/10.1021/acsomega.1c01953

[37] Lucchese, M.; Stavale, F.; Ferreira, E. H. M. et al., Quantifying ion-induced defects and Raman relaxation length in graphene. *Carbon* **2010**, *48*, 1592–1597.

[38] Bokobza, L.; Bruneel, J.-L.; Couzi, M. Raman Spectra of Carbon-Based Materials (from Graphite to Carbon Black) and of Some Silicone Composites. *C* **2015**, *1*, 77–94. https://doi.org/10.3390/c1010077

[39] Cintra, E.P.; Córdoba de Torresi, S.I.; Errien, N.; Louarn, G. Determination of the Formation of Ladder Structure in Poly(5-amino-1-naphthol) by Resonant Raman and XPS Characterization. *Macromolecules* **2003**, *36*, 6, 2079–2084. https://doi.org/10.1021/ma025811x

[40] Mochalin, V.; Osswald, S.; Gogotsi, Y. Contribution of Functional Groups to the Raman Spectrum of Nanodiamond Powders. *Chem. Mater.* **2009**, *21*, 273–279. https://doi.org/10.1021/cm802057q

[41] Santos, V.O.; Leite, I.R.; Brolo, A.G.; Rubim, J.C. The electrochemical reduction of CO_2 on a copper electrode in 1-n-butyl-3-methyl imidazolium tetrafluoroborate (BMI·BF_4) monitored by surface-enhanced Raman scattering (SERS). *J. Raman Spectrosc.*, **2016**, *47*, 674–680. https://doi.org/10.1002/jrs.4871

[42] Matsoso, B. J.; Ranganathan, K.; Mutuma, B. K.; Lerotholi, T.; Jones, G.; Coville, N. J. Time-dependent evolution of the nitrogen configurations in N-doped graphene films. *RSC Adv.*, **2016**, *6*, 106914–106920. https://doi.org/10.1039/C6RA24094A

[43] Menges, F. Spectragryph Optical Spectroscopy Software, Version 1.2.14. Available online: http://www.effemm2.de/spectragryph/ (accessed on 15 May 2022).

[44] Wojdyr, M. A curve fitting and data analysis program, Version 1.3.1. Available online: https://fityk.nieto.pl/ (accessed on 15 May 2022).

[45] Sun, Y-P.; Zhou, B.; Lin, Y.; et al. Quantum-sized carbon dots for bright and colorful photoluminescence. *J. Am. Chem. Soc.* **2006**, *128*, 7756–7757. https://doi.org/10.1021/ja062677d

[46] Liu, H.; Wang, Q.; Shen, G. et al. A multifunctional ribonuclease A-conjugated carbon dot cluster nanosystem for synchronous cancer imaging and therapy. *Nanoscale Res. Lett.* **2014**, *9*, 397. https://doi.org/10.1186/1556-276X-9-397

[47] Pan, D.; Zhang, J.; Li, Z.; Wu, C.; Yan, X.; Wu, M. Observation of pH-, solvent-, spin-, and excitation-dependent blue photoluminescence from carbon nanoparticles. *Chem. Commun.* **2010**, *46*, 3681–3683. https://doi.org/10.1039/c000114g
[48] Peng H, Travas-Sejdic J: Simple aqueous solution route to luminescent carbogenic dots from carbohydrates. *Chem. Mater.* **2009**, *21*, 5563–5565. https://doi.org/10.1021/cm901593y
[49] De, B.; Karak, N. A. green and facile approach for the synthesis of water soluble fluorescent carbon dots from banana juice. *RSC Adv*. **2013**, *3*(22), 8286–8290. doi:10.1039/c3ra00088e
[50] Zhou, Y.; Desserre, A.; Sharma, S.K.; *et al*. Gel-like carbon dots: characterization and their potential applications. *Chemphyschem* **2017**, *18,* 890–897. doi:10.1002/cphc.201700038.
[51] Zhou, Y.; Liyanage, P.Y.; Devadoss, D.; *et al*. Nontoxic amphiphilic carbon dots as promising drug nanocarriers across the blood–brain barrier and inhibitors of β-amyloid. *Nanoscale* **2019**, *11*, 22387–22397. doi:10.1002/cphc.201700038
[52] Wang, H., Sun, P., Cong, S. *et al.* Nitrogen-Doped Carbon Dots for "green" Quantum Dot Solar Cells. *Nanoscale Res Lett* **11,** 27 (2016). https://doi.org/10.1186/s11671-016-1231-1
[53] Zhang, X., Zhang, Y., Wang, Y., Kalytchuk, S.; *et al*. Color-switchable electroluminescence of carbon dot light-emitting diodes. *ACS Nano* **2013**, *7*, 11234–11241.
[54] Zheng, B.; Chen, Y., Li, P.; *et al*. Ultrafast ammonia-driven, microwave-assisted synthesis of nitrogen-doped graphene quantum dots and their optical properties. *Nanophotonics*, 2017, *6*, 259–267. https://doi.org/10.1515/nanoph-2016-0102
[55] Tang, L.; Ji, R. Li, X., et al. Deep ultraviolet to near-infrared emission and photoresponse in layered n-doped graphene quantum dots. *ACS Nano* **2014**; *8*, 6312–6320. https://doi.org/10.1021/nn501796r
[56] Tang, L.; Ji, R.; Li, X.; et al. Energy-level structure of nitrogen-doped graphene quantum dots. *J. Mater. Chem. C*. **2013**, *1*, 4908–4915. https://doi.org/10.1039/c3tc30877d
[57] Mewada, A.; Pandey, S.; Shinde, S.; et al. Green synthesis of biocompatible carbon dots using aqueous extract of Trapa bispinosa peel, *Mater. Sci. Enging. C.*, **2013**, *33*(5), 2914–2917. https://doi.org/10.1016/j.msec.2013.03.018.
[58] Huang, G.; Chen, X.; Cong Wang, C.: et al., Photoluminescent carbon dots derived from sugarcane molasses: synthesis, properties, and applications. *RSC Adv.*, **2017**, *7*, 47840–47847. https://doi.org/10.1039/C7RA09002A
